# Selective Coil Embolization of a Distal Segmental Renal Artery Lesion Causing Renovascular Hypertension

**DOI:** 10.1007/s00270-026-04500-9

**Published:** 2026-06-19

**Authors:** Jørgen Joakim Jørgensen, Kristina Gervin, Andreas Bachke, Helga Gudmundsdottir, Kjersti Baksaas-Aasen

**Affiliations:** 1 Department of Vascular Surgery, Oslo University Hospital, Oslo, Norway; 2 Department of Traumatology, Oslo University Hospital, Oslo, Norway; 3 Department of Research and Innovation, Division of Clinical Neuroscience, Oslo University Hospital, Oslo, Norway; 4 Department of Radiology, Oslo University Hospital, Oslo, Norway; 5 Department of Nephrology, Oslo University Hospital, Oslo, Norway; 6 Department of Anesthesia and Intensive Care Medicine, Oslo University Hospital, Oslo, Norway

Dear Editor,

Secondary hypertension is a potentially reversible condition, particularly in patients with abrupt onset or treatment-resistant blood pressure elevation. While atherosclerotic renal artery stenosis and fibromuscular dysplasia are the most common causes of renovascular hypertension, distal segmental renal artery lesions are rare and likely under-recognized [1]. In this setting, regional renal hypoperfusion may activate the renin–angiotensin–aldosterone system (RAAS) and result in severe hypertension [2]. Renal infarction typically presents with flank pain and hypertension and is frequently misdiagnosed due to non-specific symptoms [3].

A 46-year-old woman with no prior history of hypertension presented with persistent headache, right-sided flank pain, and severe hypertension (190–210/110–130 mmHg). Symptoms had progressed over several weeks prior to presentation, and she had been followed by her general practitioner over a four-week period with repeated consultations. Antihypertensive therapy was initiated early and progressively escalated without clinical effect, prompting hospital admission.

Her medical history included heterozygous factor V Leiden mutation, prior deep venous thrombosis, and catheter ablation in 2021 for right ventricular outflow tract ventricular ectopy and atrioventricular nodal re-entry tachycardia. No sustained arrhythmia or atrial fibrillation had been documented.

**Fig. 1:**
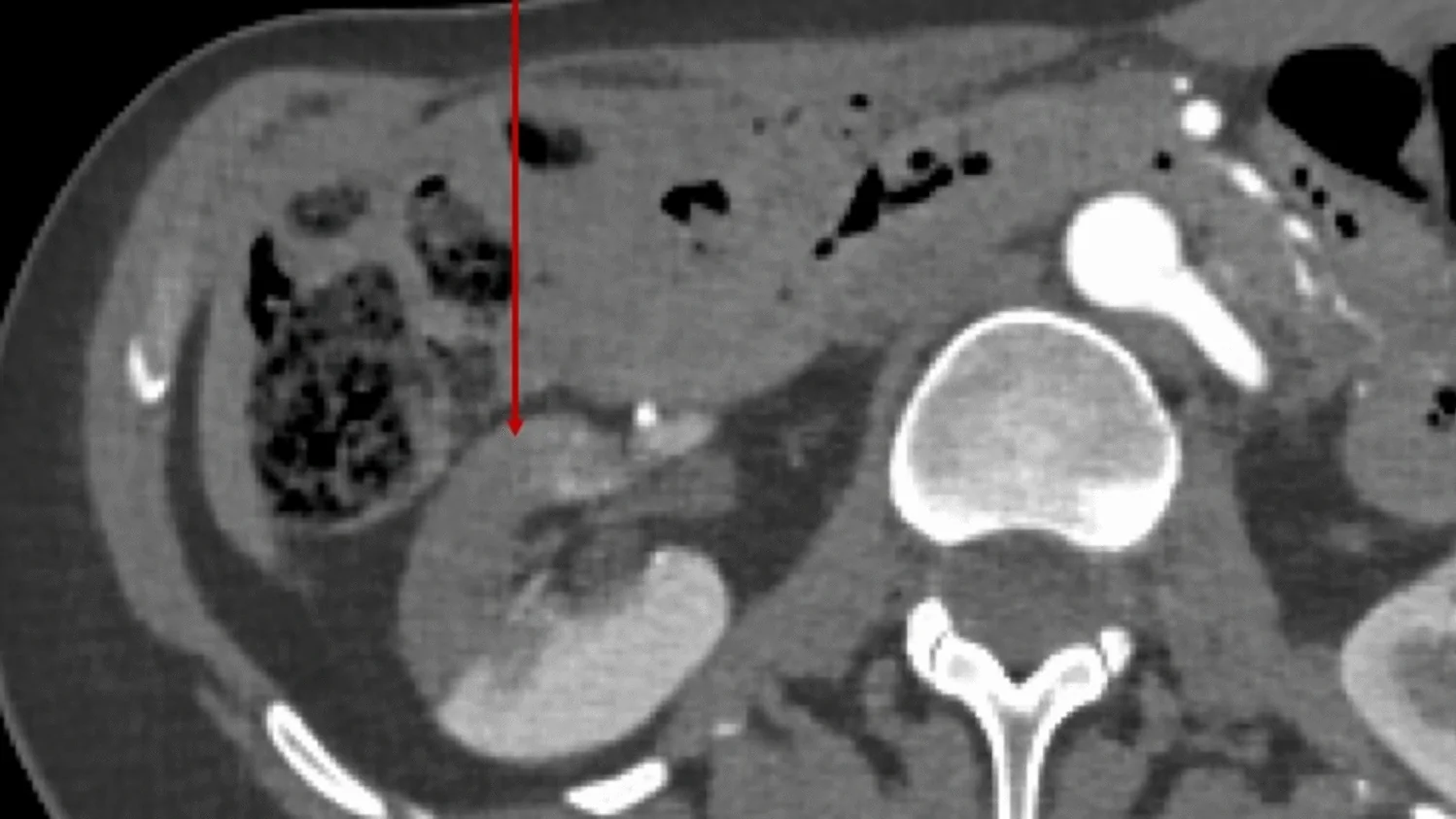
Contrast-enhanced CT demonstrating a wedge-shaped cortical perfusion defect in the right kidney (arrow), consistent with segmental renal infarction

**Fig. 2:**
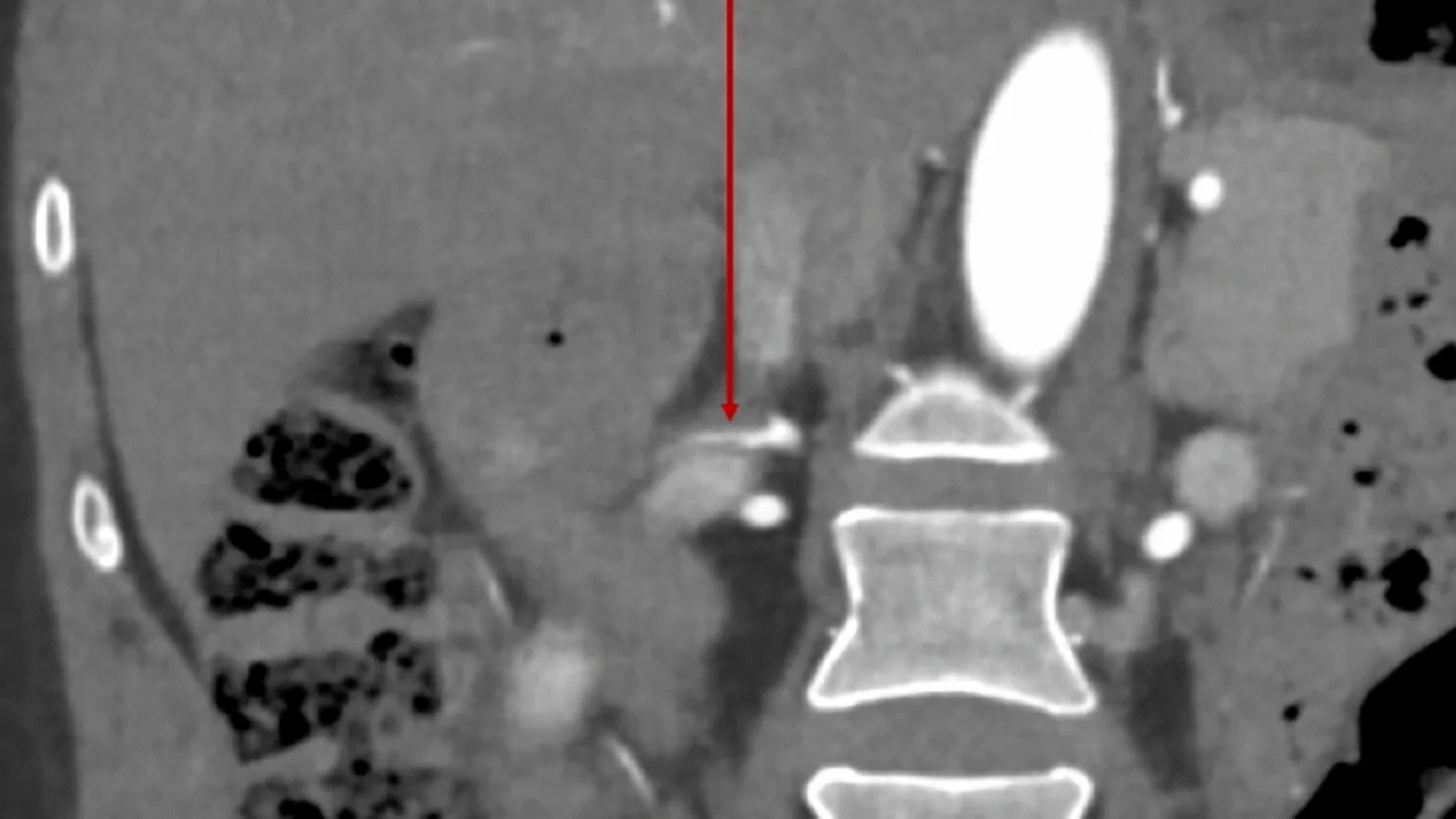
CT angiography demonstrating a focal distal segmental renal artery lesion (arrow) with luminal irregularity supplying the infarcted renal segment

Initial evaluation prioritized neurological causes. Non-contrast CT brain, brain MRI, and MR venography were normal. Due to persistent flank pain, contrast-enhanced CT angiography of the abdomen was performed, demonstrating a wedge-shaped cortical perfusion defect in the right kidney consistent with segmental renal infarction (Fig. 1), along with a focal distal segmental renal artery lesion with luminal irregularity suggestive of stenosis and/or dissection (Fig. 2). There was no evidence of generalized atherosclerosis or fibromuscular dysplasia.

After identification of renal infarction, potential embolic and inflammatory causes were systematically evaluated. Continuous telemetry showed sinus rhythm without atrial fibrillation or sustained arrhythmia, and transthoracic echocardiography revealed no intracardiac thrombus. Laboratory testing demonstrated preserved renal function, minimal inflammatory response, and negative autoimmune serology including ANA and ANCA. These findings made cardioembolic, inflammatory, and systemic etiologies unlikely.

The patient was treated with anticoagulation and multidrug antihypertensive therapy, including a renin–angiotensin system inhibitor, calcium channel blocker, thiazide diuretic, and mineralocorticoid receptor antagonist. Despite this, blood pressure remained poorly controlled and flank pain persisted.

Following multidisciplinary review, the patient was referred for endovascular evaluation. Angioplasty was not considered appropriate given the distal location, irregular morphology, and absence of viable downstream parenchyma. Because the downstream parenchyma was already infarcted and non-viable, restoring perfusion through angioplasty would not have re-established functional renal tissue, and revascularization was therefore considered physiologically futile in this setting. In addition, selective embolization allowed controlled exclusion of the ischemic segment while preserving perfusion to the remaining renal parenchyma.

**Fig. 3:**
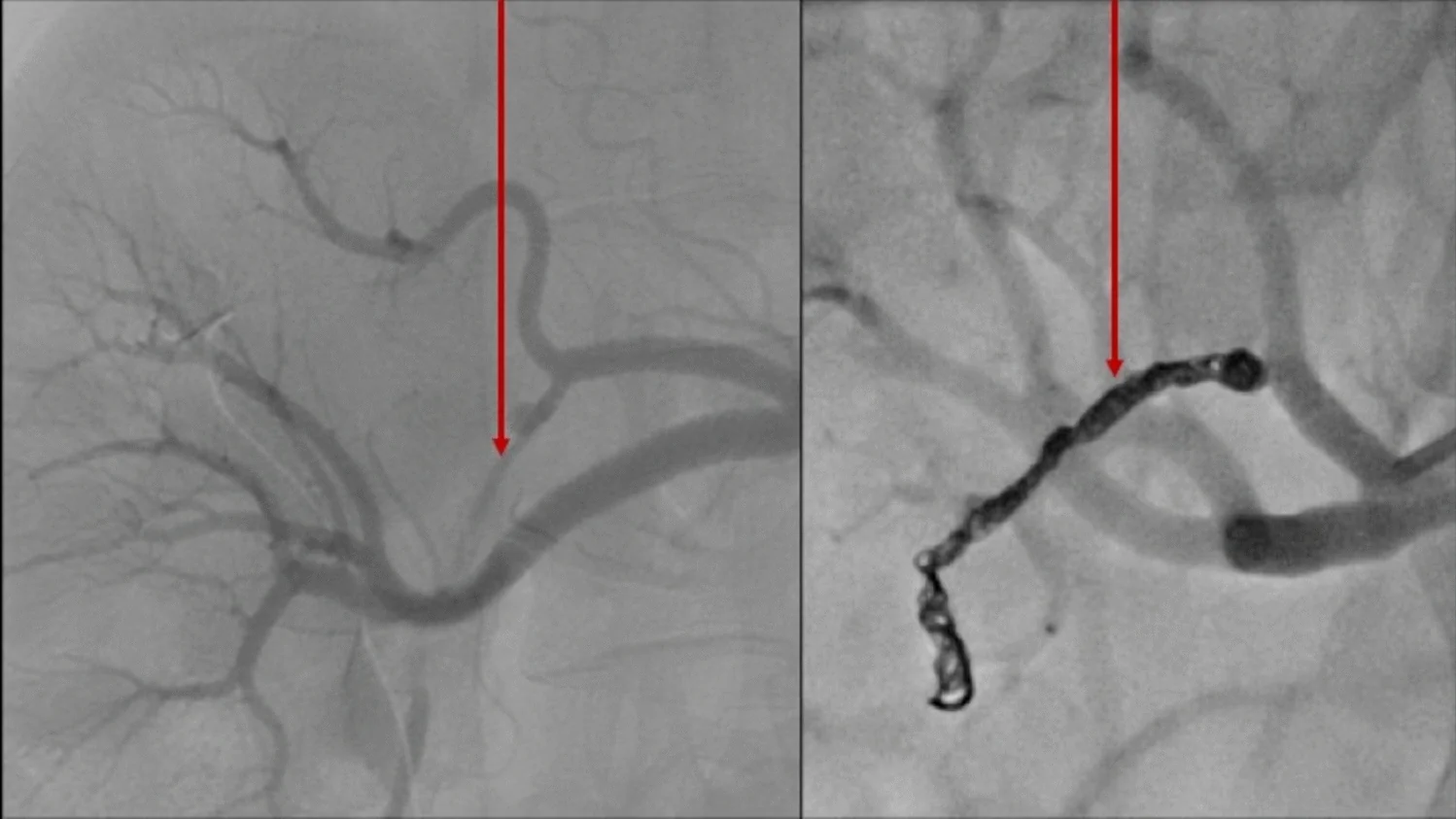
(A) Selective renal angiography demonstrating an abnormal distal segmental arterial branch supplying the infarcted territory (arrow). (B) Post-embolization angiography showing complete occlusion of the pathological branch with preserved perfusion to the remaining renal parenchyma

Selective renal angiography confirmed an abnormal distal segmental arterial branch supplying the infarcted territory (Fig. 3A). As the affected segment was non-viable and considered a likely source of renin-mediated hypertension, selective embolization was performed via transfemoral arterial access using a microcatheter system [4]. Detachable coils were deployed until complete occlusion of the pathological branch was achieved (Fig. 3B). Coils were selected to allow controlled deployment and minimize the risk of non-target embolization. N-butyl cyanoacrylate with Lipiodol is considered as an alternative embolic agent; however, coils were preferred in this case because the focal, well-delineated lesion allowed precise mechanical occlusion at a defined angiographic endpoint.

Blood pressure declined intra-procedurally and immediately following embolization. Flank pain improved within 24 h and resolved completely over the following two days. Antihypertensive therapy was progressively reduced to low-dose valsartan monotherapy. Ambulatory blood pressure monitoring demonstrated normalization (mean 128/85 mmHg) with preserved circadian variation. Renal function remained stable. At more than 12 months of follow-up, the patient remains asymptomatic and normotensive on low-dose therapy.

This case highlights a rare but clinically relevant mechanism of renovascular hypertension. Even limited areas of renal ischemia may result in disproportionate systemic effects through RAAS activation. Pharmacological therapy may attenuate downstream effects but does not eliminate the primary hemodynamic stimulus. In the present case, exclusion of the ischemic segment resulted in immediate and sustained blood pressure improvement, supporting a causal relationship. The immediate intra-procedural blood pressure reduction following occlusion of the pathological branch further supports a renin-mediated mechanism linking the ischemic segment to the patient’s hypertension.

In contrast with proximal atherosclerotic renal artery disease, distal segmental lesions represent a distinct entity. When the affected renal segment is non-viable, embolization rather than revascularization may be physiologically appropriate. Previous reports have demonstrated similar outcomes in selected cases of renin-mediated hypertension due to intrarenal branch pathology [5].

Selective embolization should therefore be considered in carefully selected patients with refractory hypertension and imaging evidence of focal renal ischemia.
